# Assessment of long-term cultivated human precision-cut lung slices as an ex vivo system for evaluation of chronic cytotoxicity and functionality

**DOI:** 10.1186/s12995-017-0158-5

**Published:** 2017-05-26

**Authors:** Vanessa Neuhaus, Dirk Schaudien, Tatiana Golovina, Ulla-Angela Temann, Carolann Thompson, Torsten Lippmann, Claus Bersch, Olaf Pfennig, Danny Jonigk, Peter Braubach, Hans-Gerd Fieguth, Gregor Warnecke, Vidadi Yusibov, Katherina Sewald, Armin Braun

**Affiliations:** 1 Fraunhofer Institute for Toxicology and Experimental Medicine, Hannover, Germany, Biomedical Research in Endstage and Obstructive Lung Disease Hannover (BREATH), Member of the German Centre for Lung Research (DZL), Member of the REBIRTH Cluster of Excellence, Hanover, Germany; 2 Fraunhofer USA Center for Molecular Biotechnology, Newark, DE USA; 3 Institute for Pathology, Hannover Medical School, Hanover, Germany, Biomedical Research in Endstage and Obstructive Lung Disease Hannover (BREATH), Member of the German Centre for Lung Research (DZL), Hanover, Germany; 4 Klinikum Region Hannover (KRH), Institute of Pathology, Hanover, Germany; 5 Klinikum Region Hannover (KRH), Division of Thoracic and Vascular surgery, Hanover, Germany; 6 Division of Cardiac, Thoracic, Transplantation, and Vascular Surgery, Hannover Medical School, Hanover, Germany, Biomedical Research in Endstage and Obstructive Lung Disease Hannover (BREATH), Member of the German Centre for Lung Research (DZL), Hanover, Germany; 7 Institute of Immunology, Hannover Medical School, Hanover, Germany

**Keywords:** Precision-cut lung slices, PCLS, Long-term cultivation, Bronchoconstriction, Human, Cytokines, Cytotoxicity

## Abstract

**Background:**

Investigation of basic chronic inflammatory mechanisms and development of new therapeutics targeting the respiratory tract requires appropriate testing systems, including those to monitor long- persistence. Human precision-cut lung slices (PCLS) have been demonstrated to mimic the human respiratory tract and have potential of an alternative, ex-vivo system to replace or augment in-vitro testing and animal models. So far, most research on PCLS has been conducted for short cultivation periods (≤72 h), while analyses of slowly metabolized therapeutics require long-term survival of PCLS in culture. In the present study, we evaluated viability, physiology and structural integrity of PCLS cultured for up to 15 days.

**Methods:**

PCLS were cultured for 15 days and various parameters were assessed at different time points.

**Results:**

Structural integrity and viability of cultured PCLS remained constant for 15 days. Moreover, bronchoconstriction was inducible over the whole period of cultivation, though with decreased sensitivity (EC_50_1d = 4 × 10^−8^ M vs. EC_50_15d = 4 × 10^−6^ M) and reduced maximum of initial airway area (1d = 0.5% vs. 15d = 18.7%). In contrast, even though still clearly inducible compared to medium control, LPS-induced TNF-α secretion decreased significantly from day 1 to day 15 of culture.

**Conclusions:**

Overall, though long-term cultivation of PCLS need further investigation for cytokine secretion, possibly on a cellular level, PCLS are feasible for bronchoconstriction studies and toxicity assays.

**Electronic supplementary material:**

The online version of this article (doi:10.1186/s12995-017-0158-5) contains supplementary material, which is available to authorized users.

## Background

Respiratory diseases, both chronic and acute, account for millions of deaths every year. In 2012 about 68% of global deaths were caused by noncommunicable diseases (NCD), with chronic lung disease being one of the four main reasons [1]. The very fact that of the approximately 38 million annual deaths attributed to NCD about 10.5% are accounted for by chronic respiratory diseases (CRD) [2], emphasizes the urgent need for improved respiratory therapies.

In this context of understanding physiological lung behavior *in-vitro* *,* it is essential to develop new therapeutic substances. In recent years, the technique of precision-cut lung slices (PCLS) has expanded the tools available for respiratory research with a link between human based in-vitro models and complex cellular anatomy [3]. PCLS, as a 3D organotypic tissue model, reflects the natural and relevant microanatomy of the respiratory tract, as well as its functional responses to specific stimuli [4–7]. Especially with regard to the three Rs principle, PCLS represents an alternative to *in-vivo* models and can help to reduce the number of animal studies [3].

Since the description of human agarose-filled lung slices in 1994 [8], optimizations have been made to reproducibly prepare very thin tissue slices of defined thickness. These improvements have led to the wide application of the PCLS technique [7, 9–11]. Ever since, PCLS from different species have been used for the assessment of pharmacological and toxicological compounds [7, 9, 10], as well as to model airway constriction [6]. Additionally, the use of PCLS as a model has expanded into the fields of nanotechnology [12, 13] and virology [14]. However, most of these studies using PCLS were conducted under short-term culturing conditions (≤72 h) [9, 13, 15], which limits their value to certain questions investigating rapid and acute effects. Accordingly, slowly metabolized therapeutics or chemicals, as well as repeated applications, which might be important to investigate basic mechanisms and treatments of CRD, have not been addressed with cultured PCLS. Even though some effort has been made prior to 2000 to establish long-term cultivation of agarose-embedded tracheal and lung tissue [16–18], these studies only focused on limited endpoints to evaluate the feasibility of long-term cultivation of lung tissue.

The maintenance of structural and cellular integrity of long-term cultivated murine or porcine lungs has primarily been evaluated microscopically [16, 17]. Recently a manuscript has been published that uses different endpoints for long-term cultivation of human PCLS, focusing on PCLS as a “high-throughput, *ex vivo* system for evaluating the safety, and potentially immunogenicity, of vaccines and pharmaceuticals” [19]. However, to our knowledge there is no study investigating multiple structural and functional endpoints after long-term cultivation of human lung tissue slices.

In the context of CRD research models, PCLS have been used as a supportive tool after disease establishment *in-vivo* [20]. This approach limits pre-clinical CRD research to either animal in-vivo studies or the use of human material from patients with the desired but very advanced disease qualifying them for lung resection.

In order to extend the research applications that PCLS can be used for, the current study evaluated long-term cultivated human PCLS with regard to their feasibility as a model for CRD research in terms of viability, physiology and structure. The intention of the study was not to introduce PCLS as a new technique, but to combine established methods and investigate the value of PCLS at a functional level as a long term cultivation model, which in our opinion has been lacking so far. The results of the present study extend the published work on long-term organ cultures and demonstrate the feasibility of using human PCLS as a long-term model for bronchoconstriction and cytotoxicity studies.

## Methods

### Culture media and reagents

Bicinchoninic acid (BCA) total protein kit, Earle’s Balanced Salt Solution (EBSS) and Dulbecco’s Modified Eagle’s Medium Nutrient Mixture F-12 Ham (DMEM, pH 7.2–7.4, without phenol red) with L-glutamine and 15 mM HEPES were obtained from GIBCO (Thermo Fisher Scientific, Rockford, IL, USA). DMEM was supplemented with 100 U/mL penicillin and 100 μg/mL streptomycin. Phosphate buffered solution (PBS, pH 7.4) and lyophilized lipopolysaccharide (LPS) of *Escherichia coli*, serotype 0111:B4 were purchased from Lonza (Verviers, Belgium). WST-1 assay kit was obtained from Roche (Mannheim, Germany). Low-gelling agarose and protease inhibitor cocktail P1860 were obtained from Sigma Aldrich (Munich, Germany).

### Human donors and ethics statement

Providing written informed consent for PCLS experiments from all patients, lung lobes were obtained immediately after surgical resection at Hannover Medical School or KRH Klinikum Oststadt-Heidehaus. For lung tumor patients, only tumor-free tissue was used. Experiments were in accordance with *The Code of Ethics of the World Medical Association* and approved by the Ethics Committee of the Hannover Medical School.

Lung donors for this study were diverse regarding age, gender, medical history and cause of resection. The average age of donors was 64 years (Additional file 1: Table S1). The number of patients is indicated in each figure.

### Preparation of PCLS

Human PCLS were prepared as previously described [7]. Briefly, lungs were gently inflated with warm 1.5% agarose-DMEM mix. Afterwards, lung explants were macroscopically assessed by an experienced pulmopathologist to identify regions of interest and exclude previously unknown medical conditions (e.g. neoplasias or infections). Sections (∅ 8 mm) were sliced in cold EBSS using a Krumdieck tissue slicer (Alabama Research and Development, Munford, AL, USA) into approx. 250–300 μm thin slices. PCLS were washed thoroughly before cultivation in DMEM (2 slice per 500 μl) under normal immersion culture conditions (37 °C, 5% CO_2_, and 100% air humidity) for up to 15 days. PCLS treated for 1 h with 1% Triton X-100 served as a dead reference.

### WST-1 assay

Viability of PCLS was assessed by metabolic activity using the WST-1 assay according to the manufacturer’s instructions and as described before [7]. PCLS were incubated with 0.125 mL/slice of diluted WST-1 solution (1:10, according to manufacture’s instructions) for 1 h at 37 °C. Optical density (OD) was measured at 420–480 nm with a reference wavelength of 690 nm.

### Calcein AM/ethidium homodimer-1 (“LIVE⁄DEAD®) staining and quantitative image analysis

For microscopic assessment of viability, PCLS were incubated with 2 μM Calcein AM and 5 μM Ethidium homodimer-I (EthD-1) for 45 min at room temperature in the dark on an orbital shaker (150 rpm) using the Live/Dead® Viability/Cytotoxicity kit from Life Technologies (Darmstadt, Germany), as previously described [7]. After washing, the slices were imaged on a confocal laser scanning microscope Meta 510 (LSM, Zeiss, Jena, Germany) using a 10× objective. From each PCLS, triplicates of 30 μm thick 3D stacks were recorded randomly and analyzed using IMARIS 7.4.0 software (Bitplane Scientific Software, Zurich, Switzerland), as described previously [7]. The ratio of counted dead cell nuclei (ex/em 517 nm/617 nm; red fluorescence) to total volume of cytoplasm of living cells (ex/em 494 nm/517 nm; green fluorescent) was calculated (dead cell nuclei/10^6^ μm^3^ cytoplasm volume).

### Measurement of cytokine secretion by enzyme-linked immunosorbent assay (ELISA)

PCLS culture supernatants were collected, supplemented with 0.2% P1860, and stored at −80 °C. Human Tumor necrosis factor-alpha (TNF-α) was measured using the human TNF-α DuoSet from R&D Systems (Wiesbaden-Nordenstadt, Germany) according to the manufacturer’s specifications and as described elsewhere [7]. OD was determined at 450 nm (reference wavelength 540 nm) using the Tecan reader Infinite 200 PRO (Crailsheim, Germany). Cytokine concentration refers to 2 slices per time point and donor, measured in duplicate.

### Methacholine-induced bronchoconstriction

Bronchoconstriction was measured according to the protocol by Seehase et al. [21]. One slice was transferred into a 6-well plate with 2 mL DMEM (supplemented with 25 mM HEPES) per measurement and fixed with a slice anchor (Warner Instruments, Hamden, CT, USA). Methacholine (Mch) was added stepwise. After addition of each concentration of Mch (10^−9^ to 10^−3^ M), pictures were recorded in 5 s intervals for 3 min with a stereo microscope (Discovery V8; Zeiss, Jena, Germany) controlled by the Axio Vision 4.8.2. software program (Zeiss, Jena, Germany). Images were analyzed with the ImageJ analysis program (National Institute of Health).

### Histopathology analysis

PCLS were fixed in 10% neutral buffered formalin, embedded in paraffin, and sliced into 4–5 μm thin slices. The paraffin slices were stained with hematoxylin and eosin (H&E) using standard histological procedures and mounted for light microscopy. Magnification and scale bars are indicated in the figure.

### Statistical analysis

Mann-Whitney tests were performed for statistical analyses using GraphPad 4.03 (GraphPad, San Diego, CA). Data were expressed as Box and Whiskers or mean ± standard error of the mean (SEM). Differences between different time points were considered statistically significant at the level of *p* < 0.05.

## Results

### Human PCLS showed persistent viability during 15-day long-term cultivation

**Fig. 1:**
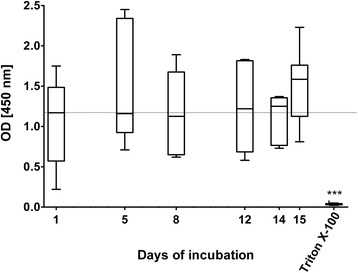
Metabolic activity of long-term cultured human PCLS. Metabolic activity in human PCLS was determined by the WST-1 assay. The negative control Triton X-100, a commonly used detergent, decreased metabolic activity in human PCLS to an OD corresponding to background (~0.04). Data are presented as *Box* and *Whiskers*, Mann-Whitney test (WST-1: *n* = 8_(day 1, 8, 15, Triton)_, *n* = 5_(day 5)_, *n* = 6_(day 12, 14)_). The dotted line marks the mean measured OD on day 1. PCLS = precision cut lung slices

In order to determine the viability of freshly prepared human lung tissue slices in immersion culture, PCLS were cultivated for 15 days and analyzed using different assays. Within this time frame no significant decrease of metabolic activity was detected by the WST-1 assay, indicating no long-term loss in viability after several days in culture. In contrast, a 1-h treatment with Triton X-100, a commonly used detergent, abrogated metabolic activity completely (Fig. 1).

**Fig. 2:**
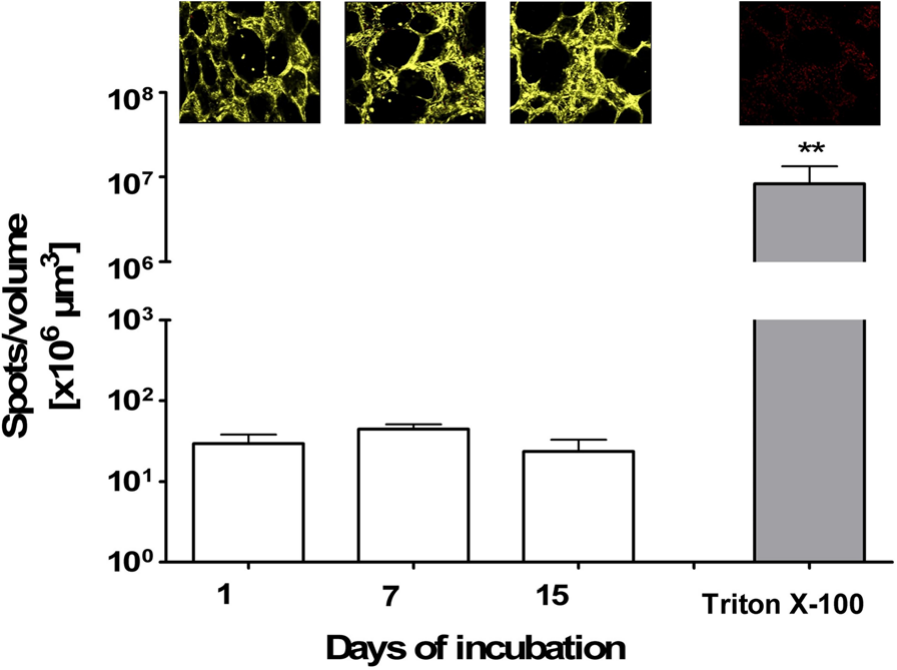
Three dimensional detection and quantitative image analysis of viability staining of long-term cultured human PCLS. Human lung slices were stained with Calcein AM (yellow) and Ethidium homodimer 1 (EthD-1; red). Three stacks per slice of 30 μm thickness were taken randomly at days 1, 7 and 15. One representative images for each time point is shown. The stacks were analysed using IMARIS 5.5.3. software and were quantitatively evaluated as the number of *red* nuclei of dead cells (*spots*) per 10^6^ μm^3^ volume of *yellow vital tissue* Triton X-100 served as a dead cell control. Data are presented as *Box* and *Whiskers*, **p < 0.01 compared to day 1, Mann-Whitney test (*n* = 6_(day 1, 7, Triton)_, *n* = 4_(day15)_)

Additionally, Calcein AM staining of the tissue showed an overall presence of living tissue with visible alveolar structures, which were maintained for the entire period of cultivation. Quantitative analysis of Live/Dead® staining showed no significant differences over time. The dead cell control with Triton X-100 showed only nuclei of dead cells and no vital tissue (Fig. 2).

### Human PCLS sustained the ability for bronchoconstriction

**Fig. 3:**
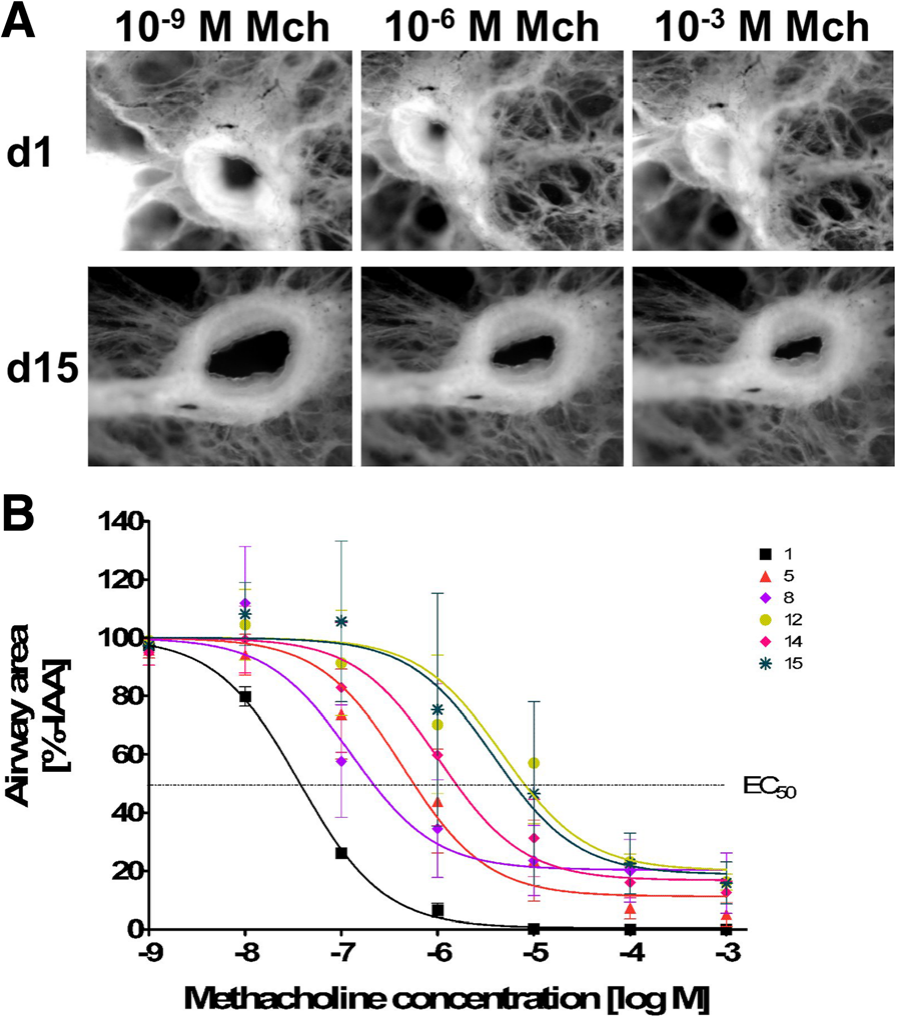
Methacholine-induced bronchoconstriction in long-term cultured human PCLS. PCLS with intact airways and surrounding epithelium were used to measure methacholine-induced bronchoconstriction at different time points. Different concentrations of methacholine (10^−9^ to 10^−3^ M) were added to the slice and slice constriction was recorded by a stereo microscope (**a**). Images were analyzed by the ImageJ analysis program. The first control picture of the non-constricted airway was set to 100%. The methacholine-induced reduction of the airway area was related to the 100% airway area of the initial airway (**b**). Measurements were conducted in at least duplicate. Data are presented as mean ± SEM, *n* = 3. Mch = methacholine, %-IAA = percentage of initial airway area, EC_50_ = half maximal effective concentration

In order to determine the physiology of long-term cultured human PCLS, Mch -induced bronchoconstriction was measured. Slices were treated with different concentrations of Mch and constriction of the airways was imaged via stereo microscopy (Fig. 3a). The ability of the PCLS to constrict upon Mch stimulus was sustained over the 15-day cultivation time (Fig. 3b). The reactivity of the airway, however, decreased during cultivation (Table 1). The capability to fully constrict upon treatment with the highest concentration of Mch, which was observed on the first day, decreased until day 15, with a maximal constriction of 81% (equals 19% initial airway area [IAA] remaining; Table 1). Additionally, the sensitivity of the airways decreased, as shown by increased EC_50_ values (Table 1). For the first 24 h after preparation, a dose of 0.37 nM Mch was sufficient to induce a 50% reduction of the IAA. In contrast, 15 days after preparation a 100-fold higher dose (38 nM Mch) was needed to achieve this same IAA reduction. A high variability between different donors, however, needs to be pointed out. Slices from different donors do not constrict equally at the same day, though the trend towards a steady decrease in reactivity and sensitivity were observed for each donor individually.

**Table 1:** Maximal airway constriction and EC_50_ concentrations of methacholine at different days of cultured human PCLS

Days of incubation	1	5	8	12	14	15
Maximal constriction [% IAA]	0.426	11	20	20	17	19
EC50 [×10^−9^ M]	0.37	4	1	47	10	38

### LPS-induced release of TNF-α was declining during 15-day long-term cultivation

**Fig. 4:**
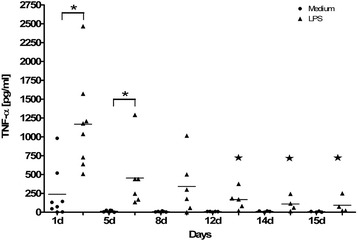
Extracellular LPS-induced release of TNF-α in long-term cultured human PCLS. Human PCLS were treated once at indicated time points without (control) or with 100 ng/mL LPS and incubated for 20 h. The cytokine levels of TNF-α in PCLS culture supernatants were determined by ELISA. TNF-α levels are depicted as mg/mL. Data are presented as mean ± SEM, *p < 0.05 compared to TNF-α secreted by untreated PCLS, ★*p* < 0.05 compared LPS-induced TNF-α at day 1, Mann-Whitney test (*n* = 8_(day 1)_, *n* = 6_(day5, 8, 12)_, *n* = 4_(day 14, 15)_). TNF-α = Tumor necrosis factor alpha, LPS = Lipopolysaccharide, ELISA = Enzyme Linked Immunosorbent Assay

The ability of PCLS to release the pro-inflammatory cytokine TNF-α upon LPS stimulation was evaluated at different time points during culture (Fig. 4). Human PCLS were stimulated once with 100 ng/mL LPS for 20 h before the released TNF-α was measured. Media-treated slices served as controls. The level of LPS-induced and secreted TNF-α was shown to continuously decrease over time, from 1168 ± 223 pg/mL at the first day post preparation to 93 ± 54 pg/mL at day 15.

However, the level of basal TNF- α measured in the untreated medium-controls also diminished drastically from 238 ± 122 pg/mL (day 1) to 13 ± 5 pg/mL (day 5) and further to 7 ± 3 pg/mL (day 15). Therefore, after 15-day cultivation, LPS-induced TNF-α secretion is still 13 times higher than the baseline level measured in the control medium (93 ± 54 pg/mL vs. 7 ± 3 pg/mL).

### The overall structural integrity of human PCLS maintained over a 15-day cultivation period

**Fig. 5:**
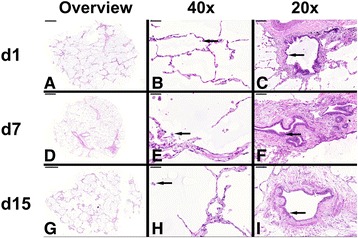
Histopathological staining of long-term cultured human PCLS. Representative pictures of human PCLS stained at days 1, 7 and 15 post preparation. The pictures show an overview of PCLS at different time points (**a**, **d** & **g**; left row), close-up pictures of alveolar regions with alveolar macrophages (**b**, **e** & **h**; arrow; middle row, 40× magnification) and close-up pictures of larger airways with bronchiolar epithelium (**c**, **f** & **i**; arrows; right row, 20× magnification)

In order to visualize pathological changes in lung tissue, PCLS were stained and imaged for histopathological analyses (Fig. 5a-i). The H&E staining revealed intact maintenance of the extracellular matrix of the lung, such as collagen and alveolar septal structure, although the slight beginning of separation of the connective tissue fibers was observed on day 7. The best preservation was observed for the bronchiolar epithelium, followed by the bronchiolar wall, the vasculature and the connective tissue. A high number of intact macrophages were observed within the tissue over time from day 1 until day 15 (Arrows Fig. 5b, e & h). The number of pneumocytes, on the other hand, seemed to be reduced during long-term cultivation. The smooth muscles remained intact for the entire period of cultivation (Arrows Fig. 5c, f & i). Also of note, there was no overgrowth of fibroblasts observable at any time point.

## Discussion

The aim of the current study was to evaluate long-term maintenance of human PCLS as a tool for CRD research, as defined by a range of viability and physiology markers. Our study completes the data set of long-term cultivation of human PCLS presented by the work of Temann et al. [19] by several important structural and functional (e.g. bronchoconstriction) assays. Overall, the present study demonstrates that human PCLS can be kept in culture for up to 15 days without the loss of structural integrity, which is consistent with earlier studies focusing only on the structure of murine tissue slices [17, 18]. Further, the here presented histology data shows no overgrowth of fibroblasts during the serum-free cultivation of human PCLS, which is in agreement with the work of Siminski et al. on mouse lungs [17]. The presented study investigates on the one hand different vitality markers, completing the recently published work of Temann et al., and on the other hand functionality markers of long-term cultivated human PCLS, which to our knowledge have not been described before. Thus, this manuscript adds useful data to evaluate the overall quality of human PCLS as a long-term tool for CRD research. For the present study, a standard slice thickness range of 250–300 μm was used in an immersion culture. In contrast, some other groups investigating long-term cultivation of lung tissue have used several millimeter thick slices and cultured them as air-liquid interface (ALI) cultures, for example either on a foam matrix or in a rotating fashion (e.g. in Leighton tubes) [16, 18]. ALI culture mimics the natural *in-vivo* air contact of the epithelial cells in-vitro [22], with one side of the tissue being exposed to about 21% oxygen. Oxygen supply is an important factor for maintaining tissue culture and the well-being of lung cells. Several years ago, an influence of tissue slice thickness and oxygen penetration was evaluated, demonstrating that increased slice thickness decreased the oxygen supply due to reduced diffusion, which subsequently leads to a decreased viability of cells in the center of the slices [23]. Although these observations were made for non-agarose filled organs different from the lung, they are conforming to the observations we made here. In line with an earlier report on long-term cultivated PCLS [19], the slice viability in the presented study was evaluated by several different assays, demonstrating a constant number of vital cells with metabolic activity over the duration of cultivation. A decreased cell vitality in the middle of the slices can mostly be excluded by microscopically Live/Dead® staining (max. Penetration depth ~ 150 μm). Application of a second assay (WST-1 in this case), however, supports the microscopic data that the tissue remains vital over the described culturing period. Additionally, microscopically observed cilia movement, as well as the ability of the airways to constrict upon Mch stimulation, both observed for the complete duration of cultivation, indicated the presence of intact, functional and vital smooth muscle and epithelial cells. This suggests a sufficient oxygen supply to PCLS, even in the immersion tissue culture.

Despite the constant viability measurements, the results of the present study also demonstrate that changes in the cellular composition and/or the functionality of certain cells may have occurred. The sensitivity of the airways to Mch stimulation decreased time-dependently (EC_50_day1 = 0.37 nM Mch vs. EC_50_day15 = 38 nM Mch). Likewise, the reactivity to Mch decreased from day 1 to day 8, as seen by the altered maximal bronchoconstriction values (IAA_day1_ = 0% vs. IAA_day8_ = 20%). However, it seems that the reactivity reached a plateau and does not further decrease after 1 week of cultivation (IAA_day8_ = 20% vs. IAA_day15_ = 19%). Therefore, a clear Mch-induced bronchoconstriction was detectable for the complete duration of the long-term cultivation.

Another alteration at the cellular level within the PCLS, which has also been described before [19], is indicated by the decreased ability to secrete the pro-inflammatory cytokine TNF-α upon LPS stimulation. Even though cells still secreted 13-times more TNF-α upon LPS stimulation at day 15 compared to the baseline level measured in the untreated controls, secretion of TNF-α decreased significantly from day 1 to day 15 (1168 ± 222.8 pg/mL vs. 93 ± 54 pg/mL). One explanation might be the serum-free or Granulocyte macrophage-colony stimulating factor (GM-CSF)-free culturing conditions used for PCLS, as TNF-α is mostly secreted by macrophages, but also by natural killer cells, B and T cells and epithelial cells [24, 25]. Macrophages in single cell culture, however, seemed to depend on the presence of GM-CSF or serum for better cell survival and TNF-α secretion upon activation [26, 27]. These macrophages secreted about twice as much TNF-α upon stimulation compared with control [26]. Noteworthy, monocytes at day 1 secreted about 4-times as much TNF-α as GM-CSF-cultivated macrophages at day 3, indicating that differentiated macrophages in culture lose TNF-α-secreting ability over time, even in the presence of GM-CSF [26]. The observations made in single cell culture systems that upon stimulation TNF-α secretion by macrophages decreases per se over time, with or without medium supplements, indicate that altered TNF-α secretion in long-term PCLS culture may be a problem of cultivation in general rather than specifically of PCLS. Further, we also observed a decline in TNF-α secretion, even for the untreated media control PCLS, from day 1 to day 5 and further until day 15 (238 ± 122 pg/mL vs. 7 ± 3 pg/mL) as has been described before [19]. This suggests that some basal TNF-α secretion occurs at the initiation of culture (day 1), either due to PCLS preparation or because of the origin of the tissue. The slicing process induces some stress for the cells, especially for those located at the cutting edge. Moreover, the complete preparation process of the human lungs (surgery, agarose filling, transport to the laboratory, cutting) takes several hours, where the lungs, though kept on ice and in media, are exposed to several stress factors. However, experience has demonstrated that after a resting phase (in medium at 37 °C) the induction of immediate early responses (e.g. genes & cytokines) are not affected [3]. In order to minimize the effect of the PCLS preparation process, the slices were thoroughly washed with medium and left for several hours under cell culture conditions before experiments were started. Yet, the influence of the cutting process, though minimized, cannot be fully excluded. It is important to note, however, that the level of this non-induced basal TNF-α release is significantly lower than what was observed after LPS treatment. In the context of a general decrease in TNF-α secretion in cell culture, it might be plausible that without the addition of essential supplements to the culture, cells responsible for TNF-α secretion may rapidly die. Yet, in the present study, macrophages appeared to survive 15-day long-term PCLS cultivation, as demonstrated by complementary histopathological analysis. However, macrophages and monocytes are not the only source of TNF-α, and other cells in PCLS may also undergo cell death in serum-free long-term culture. To elucidate this question, further studies need to look in more detail into the cellular composition of PCLS after long-term cultivation.

Working with human samples includes another important factor that needs to be considered: high inter-individual variation. Wouters et al. investigated inter- and intra-individual variations of LPS-induced cytokine (e.g. TNF-α) responses in a human whole blood assay [28]. In the present study, high inter-individual variation can be observed, especially at the first day of culture. This inter-individual variation declined during cultivation, at least for the medium control. The variations in response to LPS stimulation observed here may have been caused by the different medical background of the donors (diseases and/or different treatments, such as chemotherapy). The inter-individual variations may also play a key role in the scattering observed for the Mch-induced bronchoconstriction. Yet, regardless of the inter-individual variations, inducible bronchoconstriction and cytokine responses were observed, even after a 15-day long-term cultivation.

## Conclusions

Overall, the here presented data complement the work of Temann and colleagues [19], that long-term cultivation of PCLS for chronic exposure experiments are feasible with regard to their viability. Additionally, and new to this field is that this assumption is also supported by the maintenance of some physiological features such as bronchoconstriction. However, other questions still need to be further elucidated and protocols further adapted, to determine the exact cellular composition of lung tissue after long-term cultivation and chronic exposure. Yet, the presented data show convincingly that the extended culturing time frame of vital and functional human tissue, in regard to long-term cultivation, might increase the application of the ex-vivo technique of PCLS. PCLS have mainly been used to investigate local respiratory irritation and immunotoxicity of potential sensitizers or anti-cancer drugs [9, 29]. On the other hand, PCLS were established as disease models, reflecting features of inflammation, infection or early allergic responses mimicked by passive sensitization [14, 30–33]. Disease models involving cellular remodeling have hardly been established in PCLS. Kasper et al. established an early fibrosis model in rat PCLS using a 3-day protocol monitoring early pathohistological changes, such as extracellular matrix accumulation and myofibroblast transdifferentiation and activation of microvascular endothelial cells [34]. In line with this, Lin and colleagues also established a lung fibrosis model using 1–2 mm thick rat slices in an ALI culture, observing the fibrosis with typically increased alveolar septa thickness [35]. However, these protocols were used in rodent tissue and either did not require cultivation for longer than 3 days or did not monitor viability or functionality.

Corresponding to these earlier PCLS models of slowly progressive pulmonary diseases (e.g. interstitial lung diseases), our data might contribute to the development of further subacute or even CRD ex-vivo disease models and new therapeutic substances for these, particularly including slowly metabolized therapeutics.

## Additional file

Additional file 1: Table S1. Tissue donor information. Donor demographics including age, gender, lung lobe removed and reason for lobectomy. Yrs = Years. (DOCX 15 kb)
