# Supplementary material for: Assessment of long-term cultivated human precision-cut lung slices as an ex vivo system for evaluation of chronic cytotoxicity and functionality
Source: J Occup Med Toxicol. 2017 May 26;12:13. doi: 10.1186/s12995-017-0158-5 (PMC5446749; doi:10.1186/s12995-017-0158-5)
Supplement: Additional file 1: Table S1. — Tissue donor information. Donor demographics including age, gender, lung lobe removed and reason for lobectomy. Yrs = Years. (DOCX 15 kb) [file 12995_2017_158_MOESM1_ESM.docx]

**Additional file**

**Additional Table S1: Tissue donor information.** Donor demographics including age, gender, lung lobe removed and reason for lobectomy. Yrs = Years.

| **Number** | **Donor**  **(Gender / Age)** | **Lung lobe / Disease** | **Assay** |
| --- | --- | --- | --- |
| 1 | Male / 72 Yrs | Right upper lobe /  NSCLS | WST-1, LPS, Live/Dead staining |
| 2 | Female / 53 Yrs | left upper lobe / Graft versus Host (GvH) | WST-1, LPS, BCA, Live/Dead staining |
| 3 | Female / 63 Yrs | Right lobe / Tumor | WST-1, |
| 4 | Male / 58 Yrs | Left lobe / Fibrosis | WST-1, LPS, BCA, |
| 5 | Female / 65 Yrs | Right lobe / Adenocarcinoma | WST-1, LPS, BCA, H&E staining, |
| 6 | Female / 76 Yrs | Right lower lobe / Squamous cell carcinoma | WST-1, BCA, Bronchoconstriction, H&E staining, |
| 7 | Male / 53 Yrs | Right lobe / Emphysema | H&E staining, |
| 8 | Male / 55 Yrs | Right lower lobe / Tumor | Bronchoconstriction, H&E staining, |
| 9 | No data | Fibrosis | H&E staining, |
| 10 | Female / 66 Yrs | Right middle lobe / Adenocarcinoma | WST-1, LPS, BCA, H&E staining, Live/Dead staining |
| 11 | Female / 54 Yrs | Right upper lobe / Adenocarcinoma (with Chemotherapy) | WST-1, LPS, BCA, Bronchoconstriction, H&E staining, Live/Dead staining |
| 12 | Male / 71 Yrs | Tumor | WST-1, LPS, BCA, Live/Dead staining |
| 13 | Male / 80 Yrs | Right lobe / Adenocarcinoma | LPS, BCA, Live/Dead staining |
| 14 | Male / 71 Yrs | Tumor | Live/Dead staining |
